# Quantum-Chemistry Based Design of Halobenzene Derivatives With Augmented Affinities for the HIV-1 Viral G_4_/C_16_ Base-Pair

**DOI:** 10.3389/fchem.2020.00440

**Published:** 2020-06-19

**Authors:** Perla El Darazi, Léa El Khoury, Krystel El Hage, Richard G. Maroun, Zeina Hobaika, Jean-Philip Piquemal, Nohad Gresh

**Affiliations:** ^1^Sorbonne Université, Laboratoire de Chimie Théorique, UMR7616 CNRS, Paris, France; ^2^UR EGP, Centre d'Analyses et de Recherche, Faculté des Sciences, Université Saint-Joseph de Beyrouth, Beirut, Lebanon; ^3^SABNP, Univ. Evry, INSERM U1204, Université Paris-Saclay, Evry, France; ^4^Institut Universitaire de France, Paris, France; ^5^Department of Biomedical Engineering, The University of Texas at Austin, Austin, TX, United States

**Keywords:** HIV-1 integrase, quantum chemistry, polarizable force fields, viral DNA, Dolutegravir, INSTIs

## Abstract

The HIV-1 integrase (IN) is a major target for the design of novel anti-HIV inhibitors. Among these, three inhibitors which embody a halobenzene ring derivative (HR) in their structures are presently used in clinics. High-resolution X-ray crystallography of the complexes of the IN-viral DNA transient complex bound to each of the three inhibitors showed in all cases the HR ring to interact within a confined zone of the viral DNA, limited to the highly conserved 5′CpA 3′/5′TpG 3′ step. The extension of its extracyclic CX bond is electron-depleted, owing to the existence of the “sigma-hole.” It interacts favorably with the electron-rich rings of base G_4_. We have sought to increase the affinity of HR derivatives for the G_4_/C_16_ base pair. We thus designed thirteen novel derivatives and computed their Quantum Chemistry (QC) intermolecular interaction energies (ΔE) with this base-pair. Most compounds had ΔE values significantly more favorable than those of the HR of the most potent halobenzene drug presently used in clinics, Dolutegravir. This should enable the improvement in a modular piece-wise fashion, the affinities of halogenated inhibitors for viral DNA (vDNA). In view of large scale polarizable molecular dynamics simulations on the entirety of the IN-vDNA-inhibitor complexes, validations of the SIBFA polarizable method are also reported, in which the evolution of each ΔE(SIBFA) contribution is compared to its QC counterpart along this series of derivatives.

## Introduction

The HIV-1 integrase (IN) catalyzes the transfer of a viral DNA (vDNA) strand into the genome of the host cell (Lesbats et al., 2016). It is also involved in reverse transcription (Hironori et al., 2009), nuclear import (Mouscadet et al., 2007), and HIV-1 particle maturation (Kessl et al., 2016). It has no counterpart in human cells and thus constitutes an emerging target for the design of novel anti-retroviral inhibitors (Liao et al., 2010).

Three integrase inhibitors have been approved by the FDA in anti-HIV therapies, Raltegravir (RAL) (Summa et al., 2008), Elvitegravir (EVG) (Shimura et al., 2008), and Dolutegravir (DTG) (Underwood et al., 2012). All three act as integrase strand transfer inhibitors (INSTIs) (Ammar et al., 2016). They embody two distinct structural motives, namely a large diketo acid pharmacophore, and a halobenzene derivative. A major advance toward the design of novel derivatives is enabled by high-resolution X-ray crystal structures of the ternary complexes of IN, vDNA, and each of the three INSTI's (Hare et al., 2010a, 2011). These show all three drug complexes to be two-pronged. The keto oxygen and a coplanar neighboring oxygen both coordinate two IN catalytic Mg (II) cations, structural water molecules, and, either directly or through water, IN residues. The halobenzyl moiety is confined in a narrow cleft, binding to the G_4_ and C_16_ bases of the highly conserved 5′CpA 3′/5′TpG 3′ step on the viral DNA ends (Hare et al., 2010b). The emergence of IN mutations weakening IN-drug interactions is a major limitation for INSTI-based therapies (Wainberg et al., 2011). Such mutations were reported to occur solely on IN and not on vDNA: this thus leaves open the possibility that additional enhancements of INSTI-vDNA binding should not be adversely impacted by IN mutations.

The present study was motivated by two findings. First, reports from one of our Laboratories showed the DTG > EVG > RAL ranking of affinities for the IN-vDNA complex (denoted as the intasome, INT) to be paralleled by their corresponding affinities for the sole vDNA (El Khoury et al., 2017). Could, thus, increases of the INSTI-INT binding affinities be attempted upon focusing on the sole “ternary” complexes of G_4_, C_16_, and a halobenzene ring? Second, recent spectrometric and computational studies of the binding of INSTIs to viral DNA extremities showed the ranking of affinities to be governed by the enthalpy (ΔH) component of the binding free energies (ΔG), the entropy component (TΔS) being similar for all three complexes (El Khoury et al., 2019), a “signature” for intercalative binding (Chaires, 2006). Furthermore, the ΔH ranking of the three inhibitor affinities was itself paralleled by the corresponding ranking of the ab initio quantum chemistry (QC) intermolecular interaction energies, ΔE(QC), of their halobenzene rings with the *sole* G_4_/C_16_ base pair. Upon focusing on the halobenzene ring of the best bound compound, namely DTG, could, then, ΔE (QC) Energy Decomposition Analysis (EDA) along with electronic structure considerations offer insight for affinity-enhancing chemical substitutions?

**Figure 1:**
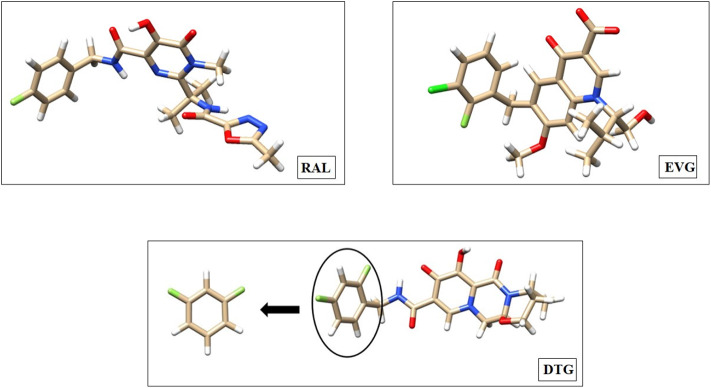
Molecular structure of Raltegravir (RAL), Elvitegravir (EVG), and Dolutegravir (DTG) represented with the Chimera software. The coordinates used for these representations are taken from the Protein Data Bank crystal structures of the Prototype Foamy Virus (PFV) intasome in complex with magnesium and Raltegravir (PDB code: 3OYA—Hare et al., 2010a), Elvitegravir (PDB code: 3L2U—Hare et al., 2010b) as well as with Dolutegravir (PBD code: 3S3M—Hare et al., 2011). We emphasized with a circle the difluoro halobenzyl ring of DTG, where the substitutions are going to be made to conceive new inhibitors. Carbon atoms are colored tan, hydrogen atoms are colored white, fluorine atoms are colored light green, chlorine atoms are colored dark green, nitrogen atoms are colored blue and oxygen atoms are colored red.

The full structures of RAL, EVG and DTG are presented in Figure 1. There is a zone of electron depletion along the extension of the C-F bond of DTG, denoted as the “sigma-hole” (Murray et al., 2008). This bond is *para* to the C-C bond connecting the ring to the central diketo acid group (Figure 1). In the crystal structure of the DTG-INT complex, it points toward the electron-rich bicyclic ring of G_4_. This could constitute a key stabilizing feature of the DTG-G_4_ complex.

We have considered and analyzed several analogs of the DTG halobenzene ring (HR), 13 of which will be reported in this paper. It is noted that with the exception of compound A1, all compounds have an extracyclic -NHCH_3_ or -NH_2_ proton donor replacing in *para* the extracyclic CF bond. All additional substitutions were done *ortho* or *para* to the C-C connector by electron-withdrawing groups. Each substituent can impact ΔE(QC) by a combination of different factors:

- on account of its electron-withdrawing character, a favorable increase of the electrostatic contribution, E_C_, of ΔE (QC), of the *para* substituent with G_4_;
- a favorable increase of the polarization contribution, E_pol_, of ΔE (QC), due to the contribution of its polarizability to the polarization energy of the ligand;
- when in *meta*, it could contribute to additional electrostatic interactions with sites belonging to G_4_ or C_16_, as reflected by E_C_ and E_pol_. There is a cone of electron-rich density around the CX bonds of halogens. Halogen substituents could enhance E_C_ if such a cone was in the vicinity of electron-deficient sites of C_16_.
- these three factors could be counteracted in some cases by increases of the short-range repulsion, E_X_, and, in the energy balances, by a larger solvation penalty than DTG, since most designed derivatives bear a more polar character than DTG.

EDA unravels the relative magnitudes of the individual ΔE (QC) contributions along the series investigated, and how each contribution can be impacted by each substituent. Optimizing the localized HR-G_4_-C_16_ interactions could contribute to a modular design of INSTIs as a preliminary to long-duration molecular dynamics (MD) of the entirety of the INSTI with the entirety of the INT. The binding site includes highly polar protein residues, two Mg (II) cations, and structural, highly polarizable water molecules. Polarizable, multipolar Molecular Mechanics/Dynamics approaches, such as SIBFA (Gresh et al., 2007) or AMOEBA (Ponder et al., 2010) should be adapted to these simulations. Such procedures were demonstrated to reliably account for the impact of the sigma hole and the dual character of the CX bond of HR on electrostatics (El Hage et al., 2013). Very large macromolecular complexes, such as the INT-INSTI ones, are now amenable to long-duration MD upon resorting to the massively parallel code Tinker-HP (Lagardère et al., 2018). Prior to these, and in the context of the present study, it was thus essential to evaluate the accuracy of one of these procedures, SIBFA. Specifically, how well will the evolutions of ΔE(QC) and of each of its contributions, be paralleled by the SIBFA contributions along the series of the thirteen HR ligands?

As a complement to EDAs, we will also report the contours of their electrostatic potential maps.

## Methods

### Quantum Chemistry Calculations

#### Energy Decomposition Analysis

The decomposition of the *ab initio* SCF interaction energy is done using the Reduced Variational Space (RVS) analysis (Stevens and Fink, 1987), where the intermolecular interaction energy is separated into four contributions: Coulomb (E_C_) and short-range exchange-repulsion (E_X_) in first order (E_1_) and polarization (E_pol_) and charge-transfer (E_ct_) in second order (E_2_). Finally, the dispersion contribution is assessed as the difference between the BSSE-corrected B97-D3 intermolecular interaction energies and the Hartree-Fock (HF) ones. The basis set superposition error (BSSE) is taken into consideration in the final energy values. The GAMESS software with the cc-pVTZ (-f) basis set (Schmidt et al., 1993) were used in this analysis. We denote below as E_pol_ (VR), a “variational” value of E_pol_, obtained as the difference between ΔE(RVS) and the sum of E_1_ and E_ct_. This is to be contrasted to E_pol_(RVS) at the outcome of the RVS procedure, the sum of all individual ligand polarization energies computed in a process when the occupied molecular orbitals (MO) of this ligand is relaxed toward its own virtual MO's, the other ligands being frozen.

Energy decomposition analyses were also performed at the correlated levels and the ω-B97-D3 functional (Grimme, 2006) resorting to the Absolutely Localized Molecular Orbitals method (ALMOEDA) (Azar and Head-Gordon, 2011) using the Q-Chem software (Shao et al., 2015). ΔE is decomposed into a “frozen density” component (FRZ), namely the sum of the Coulomb and short-range contribution, and a polarization (E_pol_) and a charge transfer contribution (E_ct_) in second order.

#### Correlated Calculations

The intermolecular interaction energies (ΔE) of the complexes formed by the substituted rings with G_4_ and C_16_ were computed at the correlated level using the dispersion-corrected functionals B97-D3 and B3LYP-D3 (Goerigk and Grimme, 2011), with the cc-pVTZ basis set (-f) (Feller, 1996) and the Gaussian software G09 (Frisch et al., 2009) software. The obtained values were corrected for BSSE (Simon and Duran, 1996). The values of the corresponding correlation and dispersion contributions were computed as the differences between the dispersion-corrected correlated ΔE and uncorrelated Hartree-Fock (HF) ΔE values.

G09 was used as well for energy-minimization of the complexes, using a starting structure determined by X-ray crystallography and taken from Protein Data Base site (PDB code: 3S3M, Hare et al., 2011). The C-C bond connecting the HR to the diketoacid (DKA) ring was replaced by a CH bond, since the DKA ring was removed.

The halobenzene rings were relaxed except for the hydrogen atom of this CH bond. This choice was made to avoid wanderings over the G4/C16 bases prevented in the complete complexes by HR anchoring to the rest of the drug. We also chose to hold both guanine and cytosine frozen in their X-ray geometry to account for their anchoring in the DNA backbone. We did not allow for conformational relaxation around the glycosidic bonds of G4 and C16 since we considered that this viral DNA base pair is held in an experimental position “tailored” for DTG: any additional positional relaxation in complexes with derivatives with bulkier ligands could *a priori* be expected to further optimize, rather than penalize, the binding of such derivatives, with possibly an even more favorable outcome than the one from the present study. This could clearly, only be clarified at the outcome of long-duration MD simulations on the ternary DNA-IN-ligand complexes, enabling to explore the widths and flexibility of the energy basins in the G_4_/C_16_/HR zone. Such studies will be reported in due course.

A Continuum desolvation energy ΔG_solv_, computed following the Polarizable Continuum Model (PCM) procedure (Mennucci et al., 2002) was also calculated for each HR ring. It was considered as an upper bound to its actual PCM desolvation energy prior to its complexation.

### SIBFA Computations

In the context of the SIBFA procedure, the intermolecular interaction energy (ΔE_tot_) is computed as the sum of five contributions: electrostatic multipolar (E_MTP_), short-range repulsion (E_rep_), polarization (E_pol_), charge transfer (E_CT_), and dispersion (E_disp_). E_MTP_ is computed with distributed multipoles (up to quadrupoles) derived from the QC molecular orbitals precomputed for each individual molecule, derived from the Stone analysis (Stone and Alderton, 1985) and distributed on the atoms using a procedure developed by Vigné-Maeder and Claverie (1988). E_MTP_ is augmented with a penetration term (Piquemal et al., 2007). The anisotropic polarizabilities are distributed on the centroids of the localized orbitals (heteroatom lone pairs and bond barycenters) using a procedure due to Garmer and Stevens (1989). E_rep_ and E_CT_, the two short-range contributions, are computed using representations of the molecular orbitals localized on the chemical bonds and on localized lone-pairs. E_disp_ is computed as an expansion into 1/R^6^, 1/R^8^, and 1/R^10^ and embodies an explicit exchange—dispersion term (Creuzet et al., 1991).

### Contours of Electrostatic Potentials

The contours of molecular electrostatic potentials (MEPs) of the HRs derived from their electronic densities were displayed by the Gaussview software, implemented in the Gaussian software (Frisch et al., 2009). The colored zones are based on their electronic densities.

## Results and Discussion

**Figure 2:**
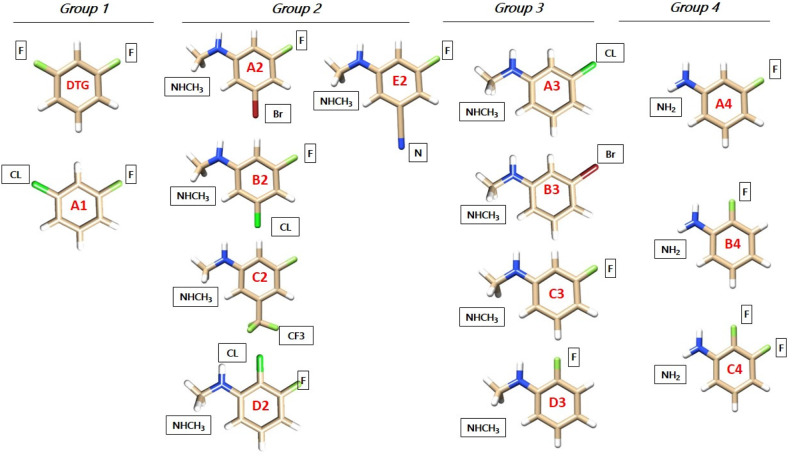
Molecular structures of the halobenzene ring of DTG (Group 1) and the newly designed ones (Groups 1, 2, 3, and 4) represented with the Chimera software. Carbon atoms are colored tan, hydrogen atoms are colored white, fluorine atoms are colored light green, chlorine atoms are colored dark green, bromine atoms are colored dark red and nitrogen atoms are colored blue. The squares indicate the new atoms or moieties chosen after substitution.

All DTG derivatives have at least one extracyclic halogen atom; F, Cl, or Br. The DTG coordinates are derived from the crystal structure of the viral intasome with Dolutegravir and two magnesium ions (PDB code: 3S3M, Hare et al., 2011). The first derivative considered, A1, has a chlorine substituent replacing the second DTG fluorine, which is *para* to the CH bond connecting it to the diketo moiety. This did not bring a significant ΔE_tot_ increase with respect to DTG (see Table 1 below). Several alternative substituents could be considered. However, for the present study we deemed it more instructive to replace upfront the *para*-F group, which binds G_4_ by its sigma-hole prolonging the CF bond, by another electron-deficient group, namely a proton donor. We could thus leverage these C-X bonds (X halogen or electron-deficient group) interactions and increase their magnitudes by depleting this donor electron-density with selected electron-attracting substituents. A natural choice bore on substituents such as -NHCH_3_ or –NH_2_. Both groups have the additional advantage of acting as electron-donating substituents: this is in contrast to fluorine, which is electron-withdrawing. This could favor the interaction of the electron-deficient sites of C_16_ with the halobenzene ring and/or with the electron-rich cone around the halogen substituent. It is another means of leveraging the “Janus-like” character of the halobenzene derivatives (El Hage et al., 2014, 2015). The –NHCH_3_ substituted derivatives were split into two groups, having two or one electron-withdrawing substituents, respectively. The search for –NH_2_ substituted derivatives was limited to three derivatives, with one or two fluorine substituents. The four groups are indexed as Group 1, 2, 3, and 4, and the derivatives in each group are indexed with capital letters from A to E. All derivatives are represented in Figure 2.

**Table 1:** Intermolecular interaction energies (kcal/mol) of halogenated derivatives with the G_4_/C_16_ vDNA base pair.

**Compounds**	**ΔE B97-D3 (raw value/BSSE corrected)**	**ΔE B3LYP-D3 (raw value/BSSE corrected)**	**ΔE ALMOEDA (B97D3)**	**ΔE ALMOEDA (ω-B97D)**	**SIBFA**	**ΔG solv**	**Total interaction energy (ΔE B97-D3 -ΔG solv) (raw value/BSSE corrected)**
**DTG**	**−37.4/−33.2**	**−40.6/−36.5**	**−33.2**	**−35.9**	**−39.6**	**−6.1**	**−31.3/−27.1**
A1	−37.5/−33.8	−40.6/−37.0	−33.8	−35.8	−38.4	−5.9	−31.6/−27.9
**A2**	**−45.1/−40.7**	**−48.1/−43.7**	**−40.6**	**−43.3**	**−45.9**	**−9.7**	**−35.4/−31.0**
**B2**	**−44.9/−40.5**	**−47.9/−43.5**	**−40.4**	**−43.1**	**−46.6**	**−9.5**	**−35.4/−31.0**
**C2**	**−46.0/−41.3**	**−49.1/−44.5**	**−41.2**	**−44.1**	**−47.3**	**−10.1**	**−35.9/−31.2**
D2	−43.0/−38.6	−46.3/−41.9	−38.5	−41.0	−45.2	−7.8	−35.2/−30.8
**E2**	**−45.3/−40.9**	**−48.2/−43.9**	**−40.6**	**−43.4**	**−47.7**	**−7.8**	**−37.5/−33.1**
A3	−44.7/−40.4	−47.6/−43.4	−40.3	−42.7	−44.6	−9.4	−35.3/−31.0
**B3**	**−44.9/−40.7**	**−47.9/−43.7**	**−40.6**	**−43.1**	**−46.9**	**−9.5**	**−35.4/−31.2**
C3	−44.1/−39.6	−47.0/−42.6	−38.8	−41.5	−45.2	−9.7	−34.3/−29.9
D3	−42.6/−37.8	−45.7/−41.1	−38.4	−41.2	−44.0	−7.8	−34.8/−30.0
A4	−42.5/−38.2	−45.2/−40.9	−38.0	−40.6	−43.7	−11.2	−31.3/−27.0
B4	−41.5/−37.0	−44.2/−39.7	−37.4	−39.7	−41.1	−9.7	−31.8/−27.3
C4	−42.1/−37.5	−45.0/−40.4	−41.2/	−41.0	−42.6	−10.3	−31.9/−27.2

*DTG as well as the compounds displaying the best energetic values are highlighted in red*.

### First Group

#### Para-Electron Attracting Substituted Derivatives

This group contains the compound A1 which has a similar structure to DTG, but with a chlorine atom in *para* instead of a fluorine one.

### Second Group

#### Para–NHCH_3_ Substituted Derivatives

Five derivatives are considered. They all have, similar to DTG, fluorine in *ortho*, and a third, electron-withdrawing substituent:

A2, B2, and C2 have in the second *ortho* position: Br, Cl, and –CF_3_, respectively. D2 has chlorine in *meta*, and E2 has a cyano substituent in the second *ortho* position.

### Third Group

#### Para–NHCH_3_ Substituted Derivatives

A3, B3, and C3 have as *ortho* substituent chlorine, bromine and fluorine, respectively, while D3 has fluorine in *meta* instead of *ortho*.

### Fourth Group

#### Para–NH_2_ Substituted Derivatives

A4 and B4 have a fluorine in *ortho* and in *meta*, respectively, while C4 has the two fluorines in *ortho* and *meta*.

**Figure 3:**
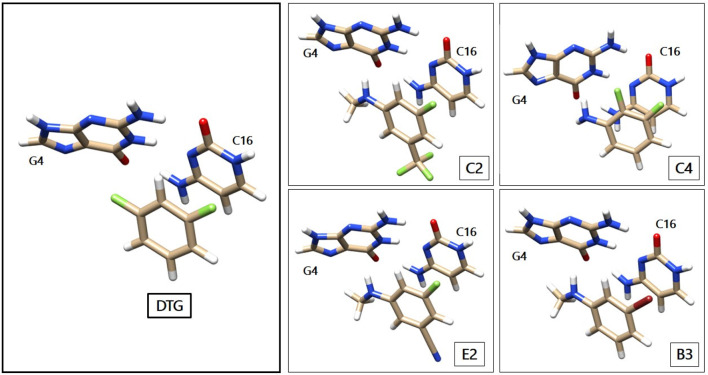
Representation of the G_4_/C_16_ complexes with representative HRs. The structures of G_4_, C_16_ and DTG are taken from the Protein Data Bank crystal structure of the Prototype Foamy Virus (PFV) intasome in complex with magnesium and Dolutegravir (PDB code: 3S3M—Hare et al., 2011). The newly designed compounds are represented with the Chimera software. The color code for the atoms remains the same as in Figure 1, with the oxygen atoms colored in light red.

Figure 3 gives a representation of the complexes of the G_4_/C_16_ vDNA base pair with representative compounds of series 1 to 4. We have displayed the parent compound, DTG from the first group, C2 and E2 from the second, B3 from the third and C4 from the fourth group. As seen below, the inhibitory rings face the ring of C_16_, interacting with the latter via π-π stacking, while the moiety in *para* position face the electron-rich rings of G_4_. Therefore, the electron donor groups in C2, E2, B3, and C4 as well as the electron-depleted region in the prolongation of the sigma-hole in DTG interacts with G_4_ via electrostatic interactions.

Table 1 lists the ΔE (QC) values at the B97-D3, B3LYP-D3, and ω-B97D levels together with the ΔE (SIBFA) values. It also lists the continuum solvation energies, ΔG_solv_, of each isolated derivative prior to complexation, and the resulting values of partial B97-D3 energy balances after ΔG_solv_ subtraction from ΔE (B97-D3). These ΔG_solv_ values should be considered as upper bounds to the actual PCM solvation energies of the halobenzene ring of DTG and its derivatives, because in the “complete” inhibitor, the exposure of the polar group is less on account of a lesser accessibility and possible intramolecular interactions. The MD calculation of the actual solvation free energies of DTG and its derivatives in a box of water molecules will be considered in the next step of our studies in parallel with the MD simulations of their complexes with the intasome (El Darazi et al., work in progress).

The listed QC ΔE values take into account the BSSE correction as the HF and at the correlated levels. It has values in the 1.5–2 kcal/mol range at the HF level, and 3.5–4.6 kcal/mol range at the correlated levels, depending upon the functional, but changing little for a given functional with the ligand.

The evolution of ΔE as a function of the compound number is plotted in Figure 3 for each functional.

**Figure 4:**
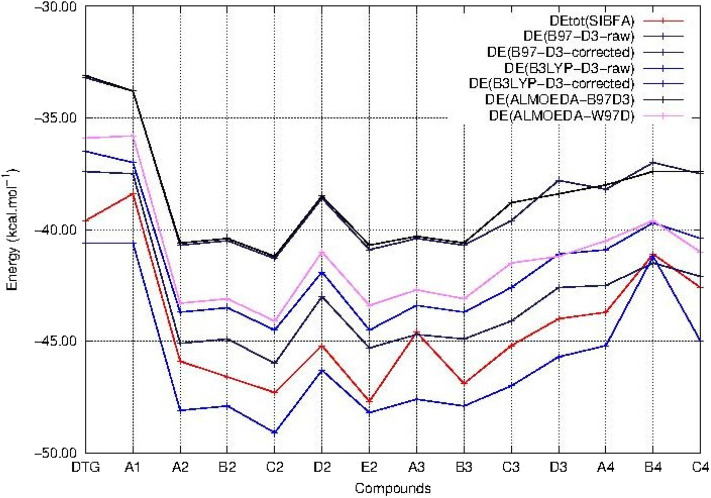
Evolution of the intermolecular interaction energies (ΔE) of the ternary complex; G_4_/C_16_/substituted ring as a function of the halobenzene ring of DTG and the newly conceived rings (Compounds A1 to C4). ΔE values are calculated via quantum chemistry correlated calculations with the B3LYP-D3 functional (ΔE B3LYP-D3-raw before BSSE correction and ΔE B3LYP-D3-corrected after BSSE correction) and the B97-D3 functional (ΔE B97-D3-raw before BSSE correction and ΔE B97-D3-corrected after BSSE correction). ΔE obtained via quantum chemistry energy decomposition analyses methods (EDA) are also presented, precisely the Absolutely Localized Molecular Orbitals method using two functionals; B97D3 (ΔE ALMOEDA-B97D3) and ω-B97D (ΔE ALMOEDA/ω-B97D). ΔE values are obtained as well via molecular mechanics calculations with the polarizable force field SIBFA (ΔE_tot_ SIBFA).

All three ΔE (QC) curves run parallel. Table 1 and Figure 4 show that the ΔE (QC) values of all compounds in series 2–4 have significantly larger magnitudes than those of either DTG or its chlorinated derivative A1. Taking the B97-D3 results as an example, the differences are in the 4–8.3 kcal/mol range. All three QC procedures concur into having five derivatives which stand out from the rest. Four belong to series 2, namely A2, B2, C2, and E2, and one belongs to series 3, namely B3. In series 2, all four derivatives have their two substituents on both *ortho* sides of the connecting -CH bond, or, equivalently, on both sides *meta* to the –NHCH_3_ para substituent. The two best-bound compounds are C2 and E2, with either a –CF_3_ or a cyano substituent –CN in *meta* to the –NHCH_3_ group, respectively. C2 has a larger ΔG_solv_ value than E2, namely −10.1 compared to −7.8 kcal/mol. As mentioned above, this 2.3 kcal/mol difference should represent an upper bound to the actual desolvation energy difference between the two derivatives. It could reflect a greater “hydrophilicity” of the C2 than the E2 ring. Including such a difference in partial relative energy balances would give rise to a 2 kcal/mol preference for the cyano derivative compared to the trifluoromethyl one.

In series 3, the best-bound derivative is B3, with a bromine substituent. Owing to the large hydrophilic character of this atom compared to fluorine in DTG, the values of ΔG_solv_ are correspondingly larger in series 2 and 3 than that of DTG, in the range 2.8–4.0 kcal/mol. This leaves out relative energy preferences for A2–C2, E2, and B3 over DTG in the range 4–6.7 kcal/mol.

The ΔE values in series 4 with an –NH_2_ para substituent have smaller magnitudes than in series 2 and 3. These are 4–5 kcal/mol more favorable than DTG, but these are virtually fully compensated for by correspondingly larger ΔG_solv_ values, leaving partial energy balances <0.5 kcal/mol more favorable than DTG, which is inconclusive in terms of augmented affinities.

As for the BSSE values, we wish to note that the average values of the BSSE amounts to 4.14 kcal/mol for the B97D-3 functional and to 3.41 kcal/mol for the W97D functional, both used for the ALMOEDA energetic calculations. With each, the relative variations among the compounds are around 0.5 kcal/mol. Whereas, at the HF level, the average value of BSSE is in the range of 1.5–2 kcal/mol.

**Figure 5:**
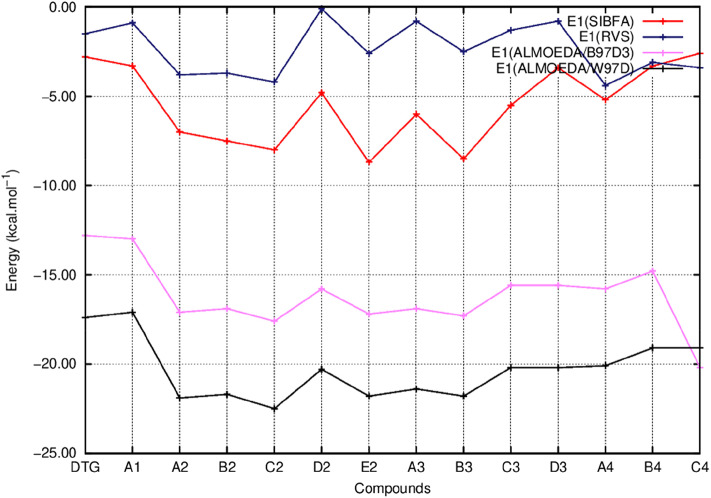
Evolution of the first order energy (E_1_) of the ternary complex; G_4_/C_16_/substituted ring as a function of the halobenzene ring of DTG and the newly conceived rings (Compounds A1 to C4). E_1_ values are calculated via quantum chemistry Energy Decomposition Analysis methods (EDA) precisely the Reduced Variational Space method (E_1_ RVS) and Absolutely Localized Molecular Orbitals method using two functionals; B97D3 (E_1_ ALMOEDA/B97D3) and ω97D (E_1_ ALMOEDA/ω-B97D). E1 values are obtained as well via molecular mechanics calculations with the polarizable force field SIBFA (E_1_ SIBFA).

**Figure 6:**
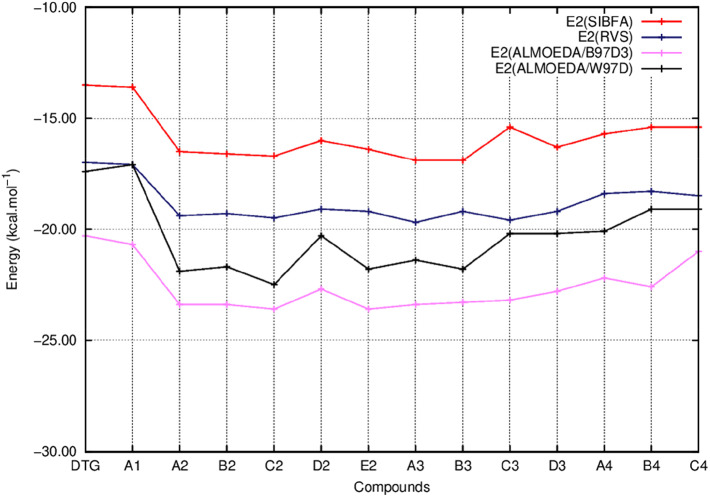
Evolution of the second order energy (E_2_) of the ternary complex; G_4_/C_16_/substituted ring as a function of the halobenzene ring of DTG and the newly conceived rings (Compounds A1 to C4). E_2_ values are calculated via quantum chemistry Energy Decomposition Analysis methods (EDA), precisely the Reduced Variational Space method (E_2_ RVS) and Absolutely Localized Molecular Orbitals method using two functionals; B97D3 (E2 ALMOEDA/B97D3) and ω-B97D (E2 ALMOEDA/ω-B97D). E_2_ values were obtained as well via molecular mechanics calculations with the polarizable force field SIBFA (E_2_ SIBFA).

**Figure 7:**
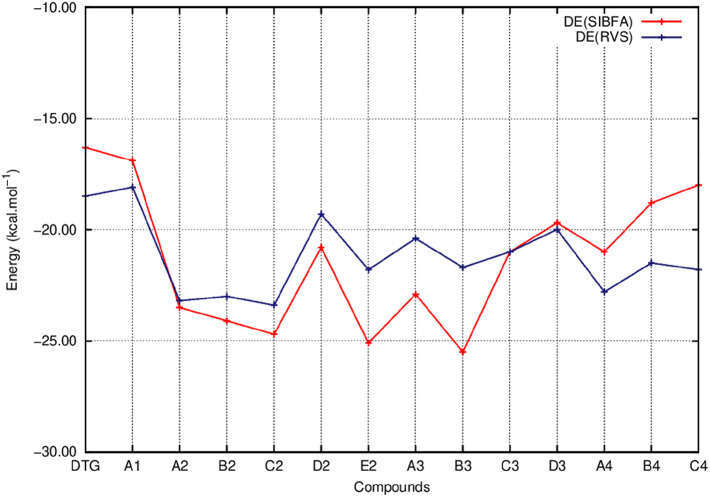
Evolution of the interaction energy of the ternary complex; G_4_/C_16_/substituted ring as a function of the halobenzene ring of DTG and the newly conceived rings (Compounds A1 to C4). ΔE values are calculated via quantum chemistry Energy Decomposition Analysis method (EDA), precisely the Reduced Variational Space method (ΔE RVS) and via molecular mechanics calculations with the polarizable force field SIBFA (ΔE SIBFA).

**Figure 8:**
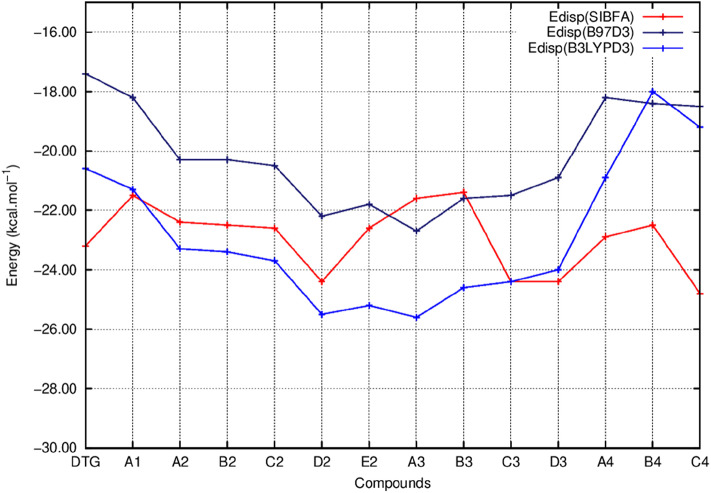
Evolution of the dispersion energy (E_disp_) of the ternary complex; G_4_/C_16_/substituted ring as a function of the halobenzene ring of DTG and the newly conceived rings (Compounds A1 to C4). E_disp_ values correspond to the ones calculated via quantum chemistry correlated calculations using two functionals; B97D3 (E_disp_ B97D3) and B3LYPD3 (E_disp_ B3LYPD3) and via molecular mechanics calculations with the polarizable force field SIBFA (E_disp_ SIBFA).

In Figures 5–**8** we report the evolution of the individual QC contributions along the series. We denote by E_1_ the “frozen” energy contribution from ALMOEDA and the sum of the Coulomb, E_C_, and exchange, E_X_, contributions from the RVS analyses. E_1_ (SIBFA) is correspondingly the sum of its penetration-augmented electrostatic and short-range repulsion contributions. We denote by E_2_ the sum of the polarization and charge-transfer contributions in all approaches.

In the perspective of long-duration polarizable MD on the complexes of vDNA and of the Integrase-vDNA assembly, it is essential to evaluate how well could a procedure such as SIBFA account for the ΔE (QC) trends. This alone could lend credence to comparative energy balances and prospective free energy calculations bearing on complexes out of reach of QC calculations, but amenable to this, and related procedures such as those between “improved” DTG derivatives with v-DNA, let alone with INT, which total several thousands of atoms. Table 1 and Figure 4 show that ΔE_tot_ (SIBFA) is fully able to recover the trends from ΔE(QC). C2 and E2 are found to be the two best-bound compounds, while B2, B3, and A2 come next with a small margin. Such agreements are encouraging, considering that no extra calibration effort was done on the DTG derivatives.

Figure 5 reports the evolutions of E_1_ at the uncorrelated RVS and correlated B97D3 and ω-B97D levels and those of E_1_ (SIBFA). Figure 6 reports the corresponding evolutions of E_2_. Figure 7 compares the evolution of uncorrelated ΔE (RVS) and ΔE (SIBFA) without the dispersion contribution E_disp_. Figure 8 reports the evolution of a contribution denoted as “E_disp_/E_corr_,” the gain in ΔE upon passing from the uncorrelated RVS ΔE to the correlated B3LYP-D3, B97-D3, and ω-B97D levels, along with E_disp_ (SIBFA). There is no explicit ALMOEDA “dispersion” contribution, as it is included in the van der Waals kernel for both E_1_ and E_2_.

Throughout we will denote by ΔE (SIBFA) and ΔE_tot_ (SIBFA) the SIBFA intermolecular interaction energies without and with the dispersion contribution. ΔE (RVS) denotes the BSSE-corrected RVS intermolecular interaction energy. ΔE (QC) denotes the correlated QC intermolecular interaction energy with the B3LYP-D3, B97-D3, or ω-B97D functionals. The QC-derived “dispersion” energy is the difference between ΔE (QC) and ΔE (HF), the latter being derived from a Hartree-Fock computation without removing the BSSE.

Figure 5 shows that the trends in ΔE (QC) and ΔE_tot_ (SIBFA) giving distinct preferences for derivatives A2, B2, C2, E2, and B3 are retrieved by E_1_, while the E_2_ curves (Figure 6) are much shallower. Figure 7 shows that similar to ΔE (QC) and ΔE_tot_ (SIBFA), both ΔE (RVS) and ΔE (SIBFA) curves have minima with derivatives A2–C2, E2, and B3. ΔE (RVS) has an additional minimum with derivative A4 from series 4. The corresponding ΔE (SIBFA) minimum is higher in energy. There is a lesser correspondence between E_disp_ (SIBFA) and those found from the B97D3 and B3LYP-D3 calculations (Figure 8). For compounds A1-E2, it has values intermediate between those from both functionals. For A3 and B3, it is close to the B97D3 values, with differences of 1.2 and 0.2 kcal/mol, respectively. For C3 and D3, it is close to the B3LYP-D3 values, with differences of 0.0 and 0.4 kcal/mol, respectively. For all three compounds in series 4 having the *para* –NH_2_ substituent, E_disp_ (SIBFA) has smaller values than either functional. In fact, the trends are not consistent between the two functionals. Thus, while E_disp_ (B3LYP-D3) is larger by 2.7 kcal/mol than E_disp_ (B97-D3), both functionals give very close E_disp_ values in both B3 and C3 complexes.

Even though it appears satisfactory, the agreement between ΔE_tot_ (SIBFA) and ΔE (QC) could be further improved in the near future. There are ongoing SIBFA refinements in the context of reconstruction of a new library of protein, DNA, and ligand constitutive fragments. They resort to multipoles and polarizabilities from correlated calculations, and a rescaling of the individual energy contributions on the basis of correlated Symmetry Adapted Perturbation Theory SAPT-DFT (Misquitta and Szalewicz, 2002; Podeszwa et al., 2006) energy decomposition analyses. They will be reported elsewhere.

### Molecular Electrostatic Potentials (MEP)

**Figure 9:**
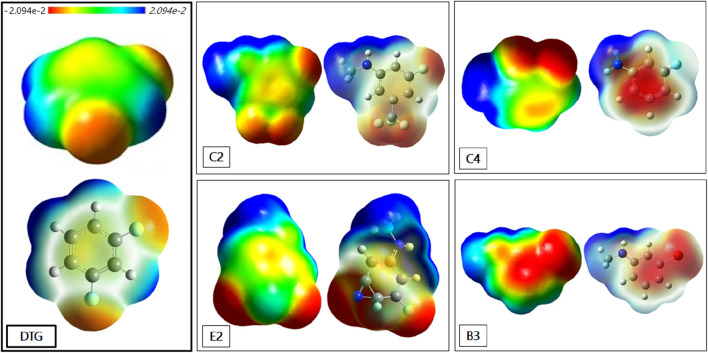
Contours of the Molecular Electrostatic Potential (MEP) around: DTG, C2, E2, B3, and C4. The distribution of the electronic charges is 10^−3^ electron Bohr-3 isodensity surface. The colored bar at the top of the figure indicates the variation of the electron density between −2.094^e−2^ (corresponding to the color red) and 2.094^e−2^ (corresponding to the color blue).

It is instructive to represent the impact of some of the reported substitutions on the MEP contours. Figure 9 presents such contours around five representative compounds: DTG; C2 and E2; the *meta-*substituted trifluoro- and cyano derivatives of group 2, respectively; B3, the *meta*-substituted bromine derivative of series 3; and C4, the difluorine-substituted derivative of group 4.

There is a much wider extension of the “blue” zone of positive potential around the *para* –NHCH_3_ or –NH_2_ substituents than around the *para*-fluorine substituent of DTG. This explains the better efficiency observed in the case of the newly conceived compounds in comparison with DTG. In fact, the lower electron density of NHCH_3_ and NH_2_ confirms the better electrostatic interaction of these rings with the electron rich polycyclic structure of G_4_. For the compounds of group 2, the –CF_3_ substituted derivative C2 generates a much wider and delocalized “red” zone of negative potential than the cyano-substituted derivative E2. The extension of positive potential around the -NHCH_3_ group is also larger for C2 than for E2. To a large extent, the first feature should explain the 2.1 kcal/mol larger ΔG_solv_ energy of C2 than E2, impacting the energy balance in favor of E2 while both derivatives had close ΔE (QC) and ΔE_tot_ (SIBFA) values. Derivative B3 also displays a wide zone of negative electrostatic potential around the –Br substituent, and this zone is significantly more extended than around the two –F substituents of derivative C4. The latter presents a low electron density in the middle of its ring due to the presence of its two-electron attracting fluorines, giving it the least favorable attraction with the DNA viral base pairs.

## Conclusions and Perspectives

We have designed a series of derivatives of the halobenzene ring of dolutegravir (DTG), the most potent inhibitor of the HIV-1 integrase to date. These derivatives target the highly conserved G_4_/C_16_ base pair of viral DNA. The -*para* fluorine ring of DTG is replaced by a –NHCH_3_ or a –NH_2_ substituent. All derivatives reported in this study had more favorable intermolecular interaction energies with this base pair than DTG, as computed by quantum chemistry, and a polarizable molecular mechanics procedure, SIBFA. The two compounds with the highest G_4_/C_16_ affinities had a *para* –NHCH_3_ substituent and two substituents *meta* to it: fluorine and trifluromethyl, denoted as C2, and fluorine and cyano, denoted as E2. ΔE_tot_ (SIBFA) displayed trends consistent with ΔE (QC). This study, along with our previous ones (El Hage et al., 2014, 2015; El Khoury et al., 2019) shows that it is possible to design, in a piece-wise fashion, novel derivatives targeting a well-defined, highly conserved, subset of the recognition site of a large macromolecular target. It also constitutes an essential validation step prior to large-scale polarizable molecular dynamics simulations of the complex of the entirety of the drug with the entirety of the target.

## Data Availability Statement

The datasets generated for this study are available on request to the corresponding author.

## Author Contributions

PD, LK, and KH performed computations. PD, RM, ZH, J-PP, and NG analyzed results. PD, J-PP, and NG wrote the paper.

## Conflict of Interest

The authors declare that the research was conducted in the absence of any commercial or financial relationships that could be construed as a potential conflict of interest.

## References

[B1] Ammar F. F. Hobaika Z. Abdel-Azeim S. Zargarian L. Maroun R. G. Fermandjian S. (2016). A targeted DNA substrate mechanism for the inhibition of HIV-1 integrase by inhibitors with antiretroviral activity. FEBS Press 6, 234–250. 10.1002/2211-5463.12025 27239438 PMC4821353

[B2] Azar R. J. Head-Gordon M. (2011). An energy decomposition analysis for intermolecular interactions from an absolutely localized molecular orbital reference at the coupled-cluster singles and doubles level. J. Chem. Phys. 132:024103 10.1063/1.3674992 22260560

[B3] Chaires J. B. (2006). A thermodynamic signature for drug-DNA binding mode. Arch. Biochem. Biophys. 453, 26–31. 10.1016/j.abb.2006.03.027 16730635

[B4] Creuzet S. Langlet J. Gresh N. (1991). Adjustment of the SIBFA method for potential maps to study hydrogen bonding vibrational frequencies. J. Chem. Phys. 88, 2399–2409. 10.1051/jcp/1991882399

[B5] El Hage K. Piquemal J. P. Hobaika Z. Maroun R. G. Gresh N. (2013). Could an anisotropic molecular mechanics/dynamics potential account for sigma hole effects in the complexes of halogenated compounds? 34, 1125–1135. 10.1002/jcc.23242 23386428

[B6] El Hage K. Piquemal J. P. Hobaika Z. Maroun R. G. Gresh N. (2014). Substituent-modulated affinities of halobenzene derivatives to the HIV-1 integrase recognition site. Analyses of the interaction energies by parallel quantum chemical and polarizable molecular mechanics. J. Phys. Chem. A 118, 9772–9782. 10.1021/jp5079899 25230384

[B7] El Hage K. Piquemal J. P. Hobaika Z. Maroun R. G. Gresh N. (2015). Could the “Janus-like” properties of the halobenzene CX bond (X- Cl, Br) be leveraged to enhance molecular recognition? 36, 210–221. 10.1002/jcc.23786 25408206

[B8] El Khoury L. El Hage K. Piquemal J. P. Fermandjian S. Maroun R. G. Gresh N. (2019). Spectrometric and computational studies of the binding of HIV-1 integrase inhibitors to viral DNA extremities. Peer J Phys. Chem. 1:e6 10.7717/peerj-pchem.6

[B9] El Khoury L. Piquemal J. P. Fermandjian S. Maroun R. G. Gresh N. Hobaika Z. (2017). The inhibition process of HIV-1 integrase by diketoacids molecules: understanding the factors governing the better efficiency of dolutegravir. Biochem. Biophys. Res. Commun. 488, 433–438. 10.1016/j.bbrc.2017.05.001 28478035

[B10] Feller D. (1996). The role of databases in support of computational chemistry calculations. J. Comput. Chem. 17, 1571–1586.

[B11] Frisch M. J. Trucks G. W. Schlegel H. B. Scuseria G. E. Robb M. A. Cheeseman J. R. (2009). Gaussian 09. Wallingford, CT: Gaussian Inc.

[B12] Garmer D. R. Stevens W. J. (1989). Transferability of molecular distributed polarizabilities from a simple localized orbital based method. J. Phys. Chem. 93,8263–8270. 10.1021/j100362a023

[B13] Goerigk L. Grimme S. (2011). A thorough benchmark of density functional methods for general main group thermochemistry, kinetics, and noncovalent interactions†. Phys. Chem. Chem. Phys. 13, 6670–6688. 10.1039/c0cp02984j 21384027

[B14] Gresh N. Cisneros G. A. Darden T. A. Piquemal J. P. (2007). Anisotropic, polarizable molecular mechanics studies of inter- and intramolecular interactions and ligand-macromolecule complexes. a bottom-up strategy. J. Chem. Theory Comput. 3, 1960–1986. 10.1021/ct700134r 18978934 PMC2367138

[B15] Grimme S. (2006). Semiempirical GGA-type density functional constructed with a long-range dispersion correction. J. Comput. Chem. 27, 1787–1799. 10.1002/jcc.20495 16955487

[B16] Hare S. Gupta S. S. Valkov E. Engelman A. Cherepanov P. (2010b). Retroviral intasome assembly and inhibition of DNA strand transfer. Nature 464, 232–236. 10.1038/nature08784 20118915 PMC2837123

[B17] Hare S. Smith S. J. Métifiot M. Jaxa-Chamiec A. Pommier Y. Hughes S. H.. (2011). Structural and functional analyses of the second-generation integrase strand transfer inhibitor dolutegravir (S/GSK1349572). Mol. Pharmacol. 80, 565–572. 10.1124/mol.111.073189 21719464 PMC3187526

[B18] Hare S. Vos A. M. Clayton R. F. Thuring J. W. Cummings M. D. Cherepanov P. (2010a). Molecular mechanisms of retroviral integrase inhibition and the evolution of viral resistance. PNAS 107, 20057–20062. 10.1073/pnas.1010246107 21030679 PMC2993412

[B19] Hironori N. Hayashi T. Takahashi T. Miyano M. Kannagi M. Masuda T. (2009). Augmentation of reverse transcription by integrase through an interaction with host factor, SIP1/Gemin2 is critical for HIV-1 infection. PloS ONE. 4:e7825. 10.1371/journal.pone.0007825 19915660 PMC2771899

[B20] Kessl J. J. Kutluay S. B. Townsend D. Rebensburg S. Slaughter A. Larue R. C.. (2016). HIV-1 integrase binds the viral RNA genome and is essential during virion morphogenesis. Cell 166, 1257–1268. 10.1016/j.cell.2016.07.044 27565348 PMC5003418

[B21] Lagardère L. Jolly L. H. Lipparini F. Aviat F. Stamm B. Jing Z. F.. (2018). Tinker-HP: a massively parallel molecular dynamics package for multiscale simulations of large complex systems with advanced point dipole polarizable force fields. Chem. Sci. 9, 956–972. 10.1039/C7SC04531J 29732110 PMC5909332

[B22] Lesbats P. Engelman A. N. Cherepanov P. (2016). Retroviral DNA integration. Chem. Rev. 116, 12730–12757. 10.1021/acs.chemrev.6b00125 27198982 PMC5084067

[B23] Liao C. Marchand C. Burke T. R. Jr Pommier Y. Nicklaus M. C. (2010). Authentic HIV-1 integrase inhibitors. Future Med. Chem. 2, 1107–1122. 10.4155/fmc.10.199 21426159 PMC3413320

[B24] Mennucci B. Tomasi J. Cammi R. Cheeseman J. R. Frisch M. J. Devlin F. J. (2002). Polarizable Continuum Model (PCM) calculations of solvent effects on optical rotations of chiral molecules. J. Phys. Chem. A 106, 6102–6113. 10.1021/jp020124t

[B25] Misquitta A. Szalewicz K. (2002). Intermolecular forces from asymptotically corrected density functional description of monomers. Chem. Phys. Lett. 357, 301–306. 10.1016/S0009-2614(02)00533-X

[B26] Mouscadet J. F. Tchertanov L. Deprez E. (2007). Structures et rôles de l'intégrase du VIH dans le cycle viral. Virologie 11, 143–152. 10.1684/vir.2007.0127

[B27] Murray J. S. Lane P. Politzer P. (2008). Expansion of the sigma-hole concept. J. Mol. Model 15, 723–729. 10.1007/s00894-008-0386-9 19082643

[B28] Piquemal J. P. Chevreau H. Gresh N. (2007). Toward a separate reproduction of the contributions to the hartree-fock and DFT intermolecular interaction energies by polarizable molecular mechanics with the SIBFA potential. J. Chem. Theory Comput. 3, 824–837. 10.1021/ct7000182 26627402

[B29] Podeszwa R. Bukowski R. Szalewicz K. (2006). Density-fitting method in symmetry-adapted perturbation theory based on kohn–sham description of monomers. J. Chem. Theory Comput. 2, 400–412. 10.1021/ct050304h 26626527

[B30] Ponder J. Wu C. Ren P. Pande V. S. Chodera J. D. Schieders M. J.. (2010). Current status of the AMOEBA polarizable force field. J. Phys. Chem. 114, 2549–2564. 10.1021/jp910674d 20136072 PMC2918242

[B31] Schmidt M. W. Baldridge K. K. Boatz J. A. Elbert S. T. Gordon M. S. Jensen J. H. (1993). General atomic and molecular electronic structure system. J. Comput. Chem. 14, 1347–1363. 10.1002/jcc.540141112

[B32] Shao Y. Gan Z. Epifanovsky E. Gilbert A. T. B. Wormit M. Kussmann J. (2015). Advances in molecular quantum chemistry contained in the Q-Chem 4 program package. Mol. Phys. 113, 184–215. 10.1080/00268976.2014.952696

[B33] Shimura K. Kodama E. Sakagami Y. Matsuzaki Y. Watanabe W. Yamataka K.. (2008). Broad antiretroviral activity and resistance profile of the novel human immunodeficiency virus integrase inhibitor elvitegravir (JTK-303/GS-9137). J. Virol. 82, 764–774. 10.1128/JVI.01534-07 17977962 PMC2224569

[B34] Simon S. Duran M. (1996). How does basis set superposition error change the potential surfaces for hydrogen-bonded dimers? J. Chem. Phys. 105:11024 10.1063/1.472902

[B35] Stevens W. J. Fink W. H. (1987). Frozen fragment reduced variational space analysis of hydrogen bonding interactions. Application to the water dimer. Chem Phys Lett. 139, 15–22. 10.1016/0009-2614(87)80143-4

[B36] Stone A. J. Alderton M. (1985). Distributed multipole analysis methods and applications. Mol. Phys. 100, 221–233. 10.1080/00268970110089432

[B37] Summa V. Petrocchi A. Bonelli F. Crescenzi B. Donghi M. Ferrara M.. (2008). Discovery of raltegravir, a potent, selective orally bioavailable HIV-integrase inhibitor for the treatment of HIV-AIDS infection. J. Med. Chem. 51, 5843–5855. 10.1021/jm800245z 18763751

[B38] Underwood M. R. Johns B. A. Sato A. Martin J. N. Deeks S. G. Fujiwara T. (2012). The activity of the integrase inhibitor dolutegravir against HIV-1 variants isolated from raltegravir-treated adults. J. Acquired Immunodefic. Synd. 61, 297–301. 10.1097/QAI.0b013e31826bfd02 22878423 PMC3804312

[B39] Vigné-Maeder F. Claverie P. (1988). The exact multicenter multipolar part of a molecular charge distribution and its simplified representations. J. Chem. Phys. 88, 4934–4948. 10.1063/1.454705

[B40] Wainberg M. A. Zaharatos G. J. Brenner B. G. (2011). Development of antiretroviral drug resistance. N. Engl. J. Med. 365, 637–646. 10.1056/NEJMra1004180 21848464

